# The Embodiment of Architectural Experience: A Methodological Perspective on Neuro-Architecture

**DOI:** 10.3389/fnhum.2022.833528

**Published:** 2022-05-09

**Authors:** Sheng Wang, Guilherme Sanches de Oliveira, Zakaria Djebbara, Klaus Gramann

**Affiliations:** ^1^Biological Psychology and Neuroergonomics, Technische Universität Berlin, Berlin, Germany; ^2^Department of Architecture, Design and Media Technology, Aalborg University, Aalborg, Denmark

**Keywords:** neuro-architecture, ecological psychology, mobile brain/body imaging (MoBI), methodology, aesthetics and ergonomics, ecological validity

## Abstract

People spend a large portion of their time inside built environments. Research in neuro-architecture—the neural basis of human perception of and interaction with the surrounding architecture—promises to advance our understanding of the cognitive processes underlying this common human experience and also to inspire evidence-based architectural design principles. This article examines the current state of the field and offers a path for moving closer to fulfilling this promise. The paper is structured in three sections, beginning with an introduction to neuro-architecture, outlining its main objectives and giving an overview of experimental research in the field. Afterward, two methodological limitations attending current brain-imaging architectural research are discussed: the first concerns the limited focus of the research, which is often restricted to the aesthetic dimension of architectural experience; the second concerns practical limitations imposed by the typical experimental tools and methods, which often require participants to remain stationary and prevent naturalistic interaction with architectural surroundings. Next, we propose that the theoretical basis of ecological psychology provides a framework for addressing these limitations and motivates emphasizing the role of embodied exploration in architectural experience, which encompasses but is not limited to aesthetic contemplation. In this section, some basic concepts within ecological psychology and their convergences with architecture are described. Lastly, we introduce Mobile Brain/Body Imaging (MoBI) as one emerging brain imaging approach with the potential to improve the ecological validity of neuro-architecture research. Accordingly, we suggest that combining theoretical and conceptual resources from ecological psychology with state-of-the-art neuroscience methods (Mobile Brain/Body Imaging) is a promising way to bring neuro-architecture closer to accomplishing its scientific and practical goals.

## A Brief Introduction: From Pre-Neuro-Architecture to Neuro-Architecture

Before the recent development of neuro-architecture as a research field (Eberhard and Gage, 2003; Eberhard, 2009b; Ruiz-Arellano, 2015), many scholars studied psychological and behavioral effects of architectural experiences in their own way. If we consider architecture as “composed structural space,” three themes that reoccur in the history of architecture practice and theory are those of *utilitas, firmitas, et venustas*, or utility, strength, and beauty (Pollio, 1914), even if this architectural triad has changed in balance and definition at different points in time. For instance, not only were the Egyptian pyramids a utility and structural achievement, but the spatial design decisions were based on beliefs about the passage from this world to the afterworld and the goal of inducing in visitors experiences related to the afterworld (Fazio et al., 2008, p. 27–33). Equally, the Greeks, who were deeply inspired by Egyptian culture (Rutherford, 2016), refined their understanding of buildings expressed in their symmetrical and pillared architecture but continued to reserve special places in the city for buildings that were considered important, such as temples. Important buildings are situated in important places, which remains a common way of building today.

Throughout the history of architecture, from Byzantine, Islamic, Medieval and Romanesque eras to Gothic, Renaissance, and Baroque architecture, the conception of architecture continuously approximated a powerful spiritual status (Fazio et al., 2008, p. 1–7). Dominating cities and important religious buildings, including churches, temples, and mosques, were carefully designed according to cultural beliefs. The implicit agreement, throughout history, seems to be that architecture, through its utility, strength, and beauty, affects the human perceiver beyond the ordinary, material world as we know it because it affects the soul and mind (Stendhal, 2010). The relation between divinity and architecture was also expressed by applying the laws of nature in spatial ratios and proportions expressed both through the facades and the plan of buildings [see e.g., Palladio (1965)]. At any rate, although design decisions about the spatial structures had for a long time been guided by metaphysical views about how the space affects the perceiver, in the nineteenth century this came to change as religion, science and technology became more independent cultural forces.

With technological advancements, such as reinforced concrete, architects began exploring how beauty emerged from the structure and utility of the building itself (Frascari, 1983; Frampton, 1985; Corbusier, 2013). Open spaces with wide-spanning beams and few structural elements commenced a turn toward the performance of the building. Statements of influential architects point to the importance of functionality for architectural design, such as Louis Sullivan^1^, Mies van der Rohe^2^, or Augustus Pugin^3^. Modern architecture has developed into an interdisciplinary field, taking advantage of the experience of other areas of science, and especially ergonomics has increasingly been reflected in modern architecture (Charytonowicz, 2000).

Modernism made one of its marks through the famous 1910 essay by Loos (2019) in which he describes how ornamentation and art had no function and were thus redundant. In European building culture, it became customary for those influenced by these ideas to see any artistic addition or ornamentation to the interior of spaces or the exterior of buildings as superfluous and to be avoided. Instead, the focus was reoriented toward the building performance, e.g., increased window sizes, bigger open spaces, rethinking city infrastructure according to means of transportation, etc. Architects would optimize the building for its conceptual function and consequently base their design decisions on how well the building would perform. The users of the building, on the other hand, have been reduced to a matter of physical proportions (Corbusier, 1954) associated with a series of assumptions on psychological and behavioral impact.

The pre-neuro-architecture belief that spatial configurations alter psychological and behavioral outcomes is clear throughout history. Designing the world meant to design human lives (including their afterlife according to the ancient Egyptians). Yet, exactly how the designed environment affects our lives remains uncovered and typically inaccessible in the writings of architects and architectural scholars. It is not the question of why we place important buildings in important places in the cities, but why we consider places to be important to begin with. If it is due to its visual exposure from within as well as from exterior vantage points, then we must acknowledge that it is based on the properties of human perception. This is precisely where neuro-architecture comes in.

### Neuro-Architecture Definition and Objectives

Neuro-architecture can be seen as an emerging field that combines neuroscience, environmental psychology, and architecture to focus on human brain dynamics resulting from action in and interaction with the built environment (Karakas and Yildiz, 2020). Some scholars also describe neuro-architecture as a field where architects collaborate with neuroscientists to scientifically explore the relationship between individuals and their surrounding environment (Ezzat Ahmed and Kamel, 2021). Regarding the rise of this discipline, the necessity of convergence among architects and neuroscientists was first mentioned in 2003 in an interview with Eberhard and Gage (2003; see also Azzazy et al., 2021). In that year, the first academic organization focusing on neuro-architecture was formed, the Academy of Neuroscience for Architecture (ANFA; Ruiz-Arellano, 2015).

According to Azzazy et al. (2021), the main objective of neuro-architecture is to study the impact of the architectural environment on the neural system. Based on the understanding of how the brain perceives its surroundings, neuroscience can improve the design process, design strategies, and inform regulations that eventually improve human health and well-being in the future (Eberhard, 2009b; Dougherty and Arbib, 2013; Azzazy et al., 2021). One of the primary foci of this framework is to investigate peoples’ experiences in various contexts, such as the role of office space design in the reduction of stress and increase in productivity, how the design of hospital rooms enhances the recovery of patients, or how the design of churches increases the sense of awe and inspiration.

### Overview of Research Paradigms and Methods in Neuro-Architecture

With the continuous development of new brain imaging technologies and new experimental paradigms over the last decades, recent neuro-architectural studies have become increasingly sophisticated. The studies can be roughly divided into two categories that either require participants to remain motionless (stationary paradigms) or that allow physical interaction with the environment (mobile paradigms). Stationary neuroimaging protocols present participants with static visual stimuli of architectural environments while they are sitting in a well-controlled laboratory or while lying in a scanner. Stationary imaging methods like magnetoencephalography (MEG), electroencephalography (EEG), or functional magnetic resonance imaging (fMRI) can reveal the neural basis of statically experiencing the built environment. While the experimental control of stationary architectural studies is often high, the ecological validity is usually low as only two-dimensional snapshots of complex three-dimensional environments are presented that do not allow any kind of interaction with the perceived environment. Mobile protocols, in contrast, allow participants to actively experience real or virtual three-dimensional artifacts with high ecological validity, at the cost of introducing noise to the recordings due to uncontrollable environments and movement-related artifacts in the few select imaging methods that are portable (Gramann et al., 2021). Thus, while stationary protocols allow for experimental control they might not be able to measure the neural aspects of humans perceiving and interacting with the built environment, rendering mobile brain imaging methods an important tool to gain deeper insights into the impact of architecture on the human experience and behavior. Together, results from both stationary and mobile brain imaging approaches can complement each other and contribute to a more comprehensive understanding of the human brain. Several studies using stationary protocols provided first important insights into the relationship of architectural design and human brain responses. These will be introduced in the next section.

## Neuro-Architecture Research Methods, Findings and Limitations

### Previous Studies in Neuro-Architecture

Most existing neuro-architectural studies are based on stationary protocols with participants focusing on visual stimuli while being seated or lying down to measure the subjective experience of architectural aesthetics. Investigating event-related potentials (ERP) of the EEG, Oppenheim et al. (2009, 2010) found that buildings that rank high regarding their social status as they are designed to be more important (like government buildings) or sublime (like religious buildings) have more impact on the perception of sublimity than low-ranking buildings (such as buildings associated with economy or the private life). In these studies, the hippocampus was shown to contribute to the processing of architectural ranking. Other studies discovered that participants perceived curvilinear spaces as more beautiful than rectilinear ones (Vartanian et al., 2013). Using fMRI, the authors explored the neural mechanism behind this phenomenon and found that when participants made approach-avoidance decisions, images of curvilinear architectural interiors activated the lingual and the calcarine gyrus in the visual cortex more than images of rectilinear interiors. When contemplating beauty, curvilinear contours activated the anterior cingulate cortex exclusively (Vartanian et al., 2013). Using the same fMRI dataset, Vartanian et al. (2015) also examined the effects of ceiling height and perceived enclosure on aesthetic judgments in architectural design. They found that rooms with higher ceilings were more likely to be judged as beautiful and activated structures involved in visuospatial exploration and attention in the dorsal stream. Open rooms were judged as more beautiful compared with enclosed rooms and activated regions in the temporal lobes associated with perceived visual motion (Vartanian et al., 2015).

While visual sensory information about architectural features directly impacts architectural experience and the accompanying brain dynamics, higher cognitive processes were also shown to provoke changes in brain activity in the context of architectural experience. For example, expectations about aesthetic value moderated people’s aesthetic judgment. Kirk et al. (2009b) found that if the same image was labeled as being sourced from a gallery rather than being computer generated, its aesthetic ratings were significantly higher. The neural mechanisms involved in this difference in aesthetic ratings were traced to the medial orbitofrontal cortex (OFC) and the prefrontal cortex (PFC; Kirk et al., 2009b). Memories and experience can also moderate architectural aesthetics judgments. This was shown by Kirk et al. (2009b) who found that architects, compared with non-architects, had increased activity of the bilateral medial OFC and the subcallosal cingulate gyrus, when making aesthetic judgments about buildings, rather than faces. These results show that expertise can modulate the response in reward-related brain areas (Kirk et al., 2009b).

While most of the above-described studies focused on the impact of architecture on aesthetic judgments and the accompanying brain dynamics, another line of research focuses on the impact of architectural designs on people’s emotional and affective state. As there are too many studies in this area to report in detail [for an overview see Higuera-Trujillo et al. (2021)], the following exemplary studies suffice to provide the reader with a broad sense of the research questions and imaging methods used in this field. For example, using EEG in a psychophysics experiment, Naghibi Rad et al. (2019) investigated the impact that window shapes in building facades had on the perceivers’ emotional state and cortical activity. Their behavioral results showed that rectangular, square, circular and semi-circular arches were considered as pleasant window shapes, while windows with triangle and triangular arches were determined as unpleasant. Regarding ERP results, the authors found that the effect of pleasant stimuli was larger in the left hemisphere than that of unpleasant ones (Naghibi Rad et al., 2019), consistent with previous notions of lateralization with regards to emotional processes (Dimond and Farrington, 1977; Reuter-Lorenz and Davidson, 1981; Canli et al., 1998). By using physiological sensors, such as EEG, Galvanic Skin Response (GSR), and eye-tracking (ET), Shemesh et al. (2021) examined the connection between geometrical aspects of architectural spaces (such as scale, proportion, protrusion, and curvature) and the user’s emotional state in expert and non-expert participants (designers and non-designers, respectively). In general, they found that large symmetrical spaces positively affect users. In addition, the more extreme a change of proportion in height P(H) or width P(W) of virtual spaces was displayed, the stronger the response of distress was observed. All physiological measurements demonstrated significantly increased signals in non-designers than those of designers. This study reflected the connection between manipulations in the geometry of the virtual space and the user’s emotional reaction, especially for non-designers (Shemesh et al., 2021). Analyzing the neural response to restorative environments to investigate stress restoration, Martínez-Soto et al. (2013) found that exposure to restorative environments (like buildings with vegetation-surrounding) led to activation of the middle frontal gyrus, middle and inferior temporal gyrus, insula, inferior parietal lobe, and cuneus. Their findings reflected that endogenous, top-down, directed attention is more active during viewing of low restorative potential vs. high restorative potential environments. This article provided empirical evidence that building-integrated vegetation could be considered for architects in order to improve stress-restoration for residents. As a last example, a study by Fich et al. (2014) found that participants immersed in an enclosed virtual room without windows exhibited greater reactivity to a stress test than those in a virtual room with windows. Physiological reactions of this stress state consisted of both heightened and prolonged spikes in salivary cortisol (Fich et al., 2014). This finding is also consistent with the conclusion of Vartanian et al. (2015), who found that participants were more likely to judge open rooms as beautiful as compared to enclosed rooms.

### Methodological Limitations of Existing Neuro-Architecture Research

A recent literature review in the field of neuro-architecture (Higuera-Trujillo et al., 2021) provided a summary of limitations of current neuro-architectural research. The first limitation, according to the authors, is that the majority of studies are confined to architectural aesthetics, not regarding other aspects of architecture like ergonomics, affordances, or functionality. Accordingly, the authors point out that it is not possible to liken architectural experience to the artistic-aesthetic experience because the latter is only one of the components of the cognitive-emotional dimension of architecture (Higuera-Trujillo et al., 2021). Combining architectural ergonomics with architectural aesthetics facilitates architectural research as it leads to a more comprehensive picture of how architecture is perceived and acted upon. That is, the utility and beauty should be investigated in combination along with the underlying neural mechanism of the user interacting with the environment.

A second limitation according to Higuera-Trujillo et al. (2021) is the low ecological validity of established brain imaging methods that come with significant restrictions regarding the mobility of the participant. Data collection in stationary participants experiencing 2D images of architectural designs come with reduced ecological validity in neuro-architecture research (Higuera-Trujillo et al., 2021). Experimental design and techniques that allow participants to freely explore their built environment will provide an ecological account of the psychological and behavioral phenomena underlying human-architecture interactions.

### New Horizons for Architectural Neuroscience

There is a demand for new research approaches to neuro-architecture expanding the horizon for neuroscience and resulting in a wider knowledge base for architecture (Eberhard, 2009a). Aligned with Eberhard’s proposition, our contention is that current neuro-architecture methodology should be compatible with ecological psychology (one of many aspects of embodied cognitive sciences) and should make use of mobile brain imaging approaches in order to overcome the above-described limitations.

Architectural experiences are embodied in the sense that people physically interact with architectural spaces while moving through a building, opening doors, or taking the stairs to perceive different perspectives of the built environment through movement (Pektaş, 2021). Therefore, the research object of neuro-architecture itself has inherent embodied features and the appropriate research methodology should also correspond to these embodied properties. In general, the proposed methodology for an ecologically more valid neuro-architecture should be in line with an architectural interaction process which is constituted by closely linked perception and action, and by an indispensable connection of our body, brain, and the environment. Architectural environments provide us with action possibilities (Jelić et al., 2016). The possibilities to act emerge from, and are automatically processed by, our brain-body system during active exploration of our surroundings.

In what follows, we first introduce the theoretical foundation of ecological psychology to then address how ecological psychology theories can be integrated with architectural principles and how the neuro-architectural research questions can be extended from aesthetics to ergonomics within an ecological psychology framework. This offers a solution to existing limitations in current neuro-architectural research. Secondly, we will introduce Mobile Brain/Body Imaging (MoBI; Makeig et al., 2009; Gramann et al., 2011, 2014) as one emerging brain imaging approach with the potential to improve the ecological validity of neuro-architecture research. By introducing representative MoBI studies, we will elucidate how the neuro-architectural research’s limitation with regards to brain imaging technique can be overcome.

## Extending the Research Question From Aesthetics to Ergonomics Using the Framework of Ecological Psychology

Ecological psychology is an embodied, situated, and non-representationalist approach to cognition pioneered by J. J. Gibson (1904–1979) in the field of perception and by E. J. Gibson (1910–2002) in the field of developmental psychology (Richardson et al., 2008; Lobo et al., 2018). Theorizing in psychology has traditionally relied on a number of dichotomies, including those of perception and action, of organism and environment, of subject and object, and of mind and body. The “ecological approach” as articulated by Gibson offers an alternative way of understanding psychological phenomena that challenges these concepts and categories. One illustration of this anti-dualism is evident in the name of the approach. Ecology is the branch of biological science concerned with understanding the relations that biological organisms bear to other organisms and to the environment. The Gibsonian approach is “ecological” because, in contrast with the idea that psychology studies the organism (i.e., its mind and behavior), it instead sees relations between organism and environment as the proper level of analysis: in this view, understanding the organism-environment system as a whole is the starting point for understanding mind and behavior (see e.g., Michaels and Palatinus (2014)).

Following from this, another dichotomy rejected in the ecological approach is the one between perception and action. As it is usually conceived, perception is an “indirect” process in which meaning is attached to otherwise meaningless or ambiguous sensory information via “detailed internal representations” (Handford et al., 1997; Craig and Watson, 2011; Rogers, 2017); or as the prominent cognitive scientist David Marr put it, “vision is the process of discovering from images what is present in the world and where it is” (Marr, 1982, p. 3). Importantly, in this understanding of perception as a matter of internally reconstructing the external world, perception is also seen as distinct and independent from action: moving around can change the input for perception, but it does not significantly alter the perceptual process itself. Ecological psychology challenges this view by treating perception and action as mutual, reciprocal, continuous and symmetrically constraining processes (Warren, 2006; Richardson et al., 2008; Heras-Escribano, 2021). In the Gibsonian view, perception isn’t merely associated with action, but it is an action, a process of active exploration of the environment: “perceiving is an act, not a response, an act of attention, not a triggered impression, an achievement, not a reflex” (Gibson, 1979, p. 149). As a result, in contrast with the description of the visual system as extracting information about the external world from images, Gibson proposed that the visual system is itself constituted by eyes “set in a head that can turn, attached to a body that can move from place to place” (Gibson, 1979, p. 53). And besides being inherently active, perception is also for action—a claim that is central to the Gibsonian theory of affordances.

### Affordances

“Affordance” is the term that Gibson (1966; 1977; 1979) coined to refer to the possibilities for action that the environment offers to a given organism or agent. For example, a chair affords sitting on, a cup affords grasping with one hand and drinking from, and a table affords supporting the cup. For Gibson, we don’t simply perceive chairs, cups and tables as such (i.e., as mere material objects), but rather we perceive the opportunities for action that those objects make possible for us. It is in this sense that, in the ecological view, perception is for action: perception is of affordances. Importantly, however, affordances are not properties of the objects in and of themselves. The uses and meaning that objects have (i.e., their affordances) are relative to some organism or other. For instance, in the examples just given, the cup only affords grasping and holding for agents that have opposable thumbs (or their functional equivalent); for other organisms, the cup affords different uses, including hiding behind or inside (e.g., for an insect) and a place within which to grow (e.g., for a plant, if the cup is used as a vase). Similarly, the chair affords sitting on, and it also affords stepping on (e.g., to change a lightbulb), but only for people of a certain height: for others (e.g., babies) the chair might afford hiding under or support for standing up, but it might be too tall for other uses.

It is for reasons such as these that affordances have been traditionally understood as relational or agent-relative properties: affordances are “relations between the abilities of organisms and features of the environment” [Chemero, 2003, p. 189; see also Chemero (2011)]. In a landmark study that provided early support for this relational understanding of affordances, Warren (1984) found that the boundary between climbable and unclimbable stairways corresponds to a fixed ratio between riser height and leg length. That is, instead of the stairway having the affordance of “climbability” on its own, the affordance is rather a relational property, and one that participants in Warren’s study were found to be perceptually sensitive to Warren (1984). This research provided a methodology called intrinsic measurement to quantify affordances, since the unit of climbability is not an extrinsic unit such as centimeters, but the unit intrinsic to the body-environment relation that depends on leg lengths (Warren, 1984). In a follow-up study Warren and Whang (1987) found similar results for the visual guidance of walking through apertures like doorways or other gaps on a wall: consistent with the findings from the study on stairways, an aperture’s passability was found to correspond to an objective body-scale ratio (i.e., a relational property) that is visually perceivable (Warren and Whang, 1987).

Other studies have shown that our perceptual access to such action boundaries fixed at body-scale ratios is not static, but can change over time with changes in body-scale: this varies from the short-term effect that wearing a tall wooden block under one’s shoes has on the perception of opportunities for sitting and stair climbing (Mark, 1987) up to comparatively longer-term effect of bodily changes during pregnancy on (the perception of) the passability of apertures (Franchak and Adolph, 2014). Interestingly, some of these and other studies have found that participants were wildly inaccurate when asked to estimate absolute properties (such as heights and widths in centimeters or inches), which suggests that the perception of affordances (i.e., agent-relative properties) is more fundamental than, and independent from, the perception of non-agent-relative properties.

As these examples illustrate, the concept of affordance undermines the dichotomy of perception and action because, in this view, perception is the active exploration of opportunities for action in the environment (i.e., affordances). Moreover, the ecological theory of affordance perception also illustrates the rejection of the dichotomies between organism and environment, subject and object: as relational properties, affordances are features of an organism-environment system as a whole rather than characteristics of the environment and environmental objects on their own. And insofar as affordances constitute the action possibilities that an object or the environment offers some agent, the ecological approach also challenges traditional separations between mind and body. In this view the functional “meaning” of an object does not belong to an immaterial mental dimension separate from the material dimension of the body, as if the mind has to interpret sensory stimulation in order to infer what might be possible to do: rather, affordances are the action opportunities that objects have for some agent (and that the agent can directly perceive) precisely because of the agent’s particular physical structure and bodily activity.

Through embodied experience in architectural spaces we thus encounter possibilities for action that are linked to affective, cognitive, and physiological responses. In this sense, architecture shapes the way we perceive the environment. This should change the view on how architecture influences brain dynamics. Moreover, Warren’s (1984) research can be considered as an exemplary case to combine affordances with ergonomics in an architectural environment. The intrinsic measurement of this study demonstrates that research questions on ergonomic dimensions in architecture can be raised at the ecological scale allowing for a better understanding of the user’s interaction with the architectural environment in terms of complementarity between subjective capacities and objective properties. For instance, inspired by the above studies (Warren, 1984; Mark, 1987; Warren and Whang, 1987; Franchak and Adolph, 2014), in neuro-architectural research the operationalization of experimental variables with regards to architectural affordances should take into account both environmental properties (such as the height of stairs, the size of the apertures, etc.) and participants’ physical capabilities (such as the height of legs, the width changes of the body during pregnancy, etc.). It is promising to investigate this complementarity between architectural properties and the users’ embodied abilities at the ecological scale and also its underlying brain dynamics. In addition, it demonstrates the potential of neuro-architectural research questions to be extended from aesthetics to ergonomics within an ecological psychology framework.

### Active Exploration

As just seen, according to ecological psychology agents perceive affordances in a direct process of embodied activity: it is through the agents’ active exploration of the environment (Michaels and Carello, 1981; Heft, 1989; Rietveld and Kiverstein, 2014) that affordances are perceived, rendering the embodied experience of the built environment a perception-action loop. While in the last section we described how affordances impact active exploration, we now turn to the impact of active exploration on affordances.

**FIGURE 1:**
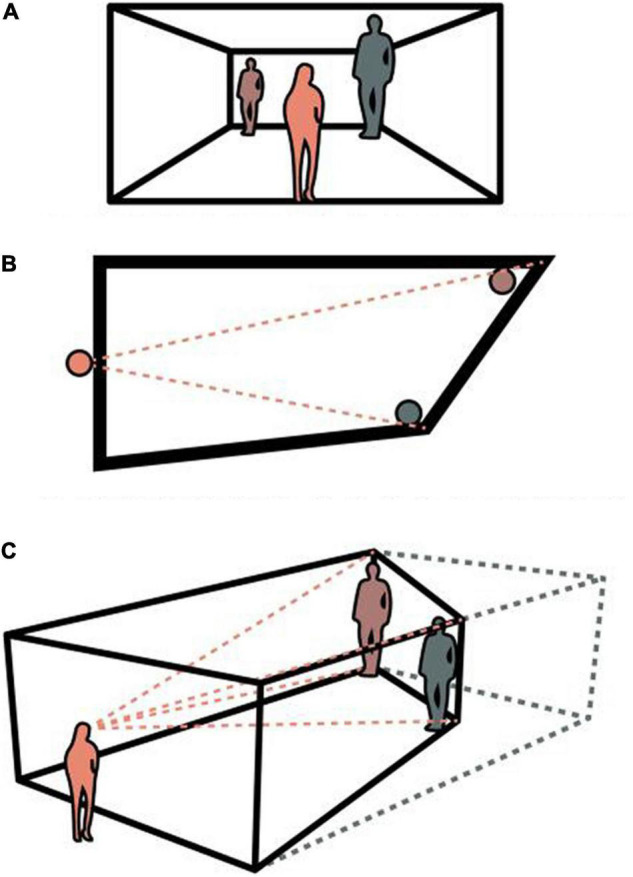
A sketch of the Ames Room. **(A)** Displays what the perceiver encounters from a given point. Color-codes are used throughout the diagrams. **(B)** Displays a conceptual plan-drawing of an Ames Room. The red dashed lines represent the field of view of the perceiver. **(C)** Reveals the actual conditions under which an Ames room functions. The red dashed lines represent the field of view of the perceiver, while the blue dashed lines represent the outline of a rectangle, which the Ames room illusion suggests to exist from a specific angle **(A)**.

Architectural affordances are perceived directly when we move through the built environment. When the observer remains stationary, or when architecture is presented as an image, architectural affordances will be limited to this one specific perspective (Heft, 2010). As stated by Heras-Escribano (2019), all organisms perceive affordances directly on the condition of unrestricted exploration and sufficient ecological information in their environment. The significance of active exploration is not only reflected in the process to discover new affordances, but also in the process of modifying existing perceptual information. The popular optical illusion of the Ames room (see Figure 1; Ittelson, 1952) was discussed by Gibson to demonstrate that the illusion could be reduced through unrestricted exploration (Gibson, 1979). Under a single and stationary point of observation of the Ames Room, the eye of the observer is fooled. When an observer views the Ames Room from various angles with binocular information, however, it is easy to notice the sharp sloped floor of the room. Normally, the ceiling and floor are parallel and walls are at a right angle to the ground; but when looking into the Ames Room, the observer can only assume that the room is geometric if active exploration is restricted. Once the observer discovers the abnormal conditions of the Ames room through active exploration, the observer will immediately reject their earlier assumption and also the existing illusory impression (Gibson, 1979). In short, the exploratory activity is crucial for both picking up new affordances and modifying existing ones. Therefore, active exploration is the core ecological approach for investigating an agents’ perception of architectural affordances.

### The Convergence of “Exploration” and “Affordance” With Architectural Design

Ecological psychology provides us with a relational perspective to account for perception and action: perception is for action, and action is for perception. This perception-action loop is neither understood as an organism-only nor an environment-only scale, but as co-depending between organism and environment. As affordances of most environments have been designed either by ourselves (e.g., our private spaces) or by architects (e.g., public spaces), we briefly investigate how architectural affordances relate to active exploration. Providing examples of ergonomic dimensions of architectural experience, the following illustrations demonstrate the convergence of “exploration” and “affordance” with architectural design.

Affordances and active exploration are not only theoretical tenets of ecological psychology, but a practical requirement of architecture: after all, every built environment, whether natural or virtual, has affordances. Instead, we focus on features of architecture that have an inviting affordance that appeals to the physical structure of the organism and its immediate relation. Carlo Scarpa, an Italian architect, was famously known for his capacity to address the rhythm of the body by creating details that invited certain movements in a specific order. *Giardino Querini Stampalia* (1961–1963) uses strategic changes in the pavement from grass, to small cobblestones and concrete, to intentionally alter the velocity of the walking, moreover all stairs in the garden have each a step for either the right or the left foot [see e.g., Dodds (2000)]. This eventually also causes different heights between steps which now also invites sitting. The rhythm and affordances of walking have then been designed by confining the actively exploring body in this case to both the velocity of the walkability and the specific order of movement for the climbability of the stairs. The very same applies to the staircase of Scarpa’s *Olivetti Showroom* (1958). As some of the steps are stretched so they float mid-air, they afford being used as a table or a place to sit (Carter, 2018).

As a second contemporary example, consider the work of RAAAF who explicitly attempts to design the affordances of the environment to make the spaces more suitable for the designed function. Consisting of the ecological psychologist and philosopher Erik Rietveld and the architect Ronald Rietveld, the duo has produced numerous projects that demonstrate how architectural affordances can inherently be used to alter the behavior of users. For instance, the project *The End of Sitting* (2014) radically challenged the mainstream structure of office landscapes by altering the affordances of “working at a desk” (Rietveld, 2016). Instead, RAAAF designed a physical landscape that invites various body postures suitable while working, e.g., laying, leaning, semi-crouching, and so on. Through active exploration, the users would realize that each part of the landscape provided its unique affordances. These examples all share inviting/suggestive designs that couple the agent with the environment in ways that alter neurobehavioral states.

These are only two of many cases in architecture in which a design principle with regards to active exploration and affordances were applied. We believe that since active exploration and affordances constitute our perception of the environment, including architectural design, any serious investigation of the experience of architecture must provide an active interaction with the environment under investigation. This view raises an important challenge for the field of neuro-architecture: studying the cognitive and neural basis of the effect of architectural features requires an interactive neuroimaging approach. In the next section, we demonstrate one way of overcoming this challenge.

## Mobile Brain/Body Imaging as a Practical Basis for Architectural Neuroscience

Mobile Brain/Body Imaging is an emerging brain/body imaging method which allows for investigating the exploratory proposition of ecological psychology with the potential to improve the ecological validity of empirical research (Parada and Rossi, 2021). Several studies in the last few years demonstrated that MoBI can be used to specifically improve the ecological validity in neuro-architectural studies by allowing for active exploration of the built environment (Banaei et al., 2017; Djebbara et al., 2019, 2021). In this section, we will describe how MoBI can improve the ecological validity of research within the field of neuro-architecture providing a brief introduction to the methods and a review of representative studies in the field of neuro-architecture.

### Mobile Brain/Body Imaging: Definition, Main Goals and Instruments

Mobile Brain/Body Imaging is defined as a multimethod approach to imaging brain dynamics in humans actively moving through and interacting with the environment (Jungnickel et al., 2019). It requires adequate hardware and software solutions to simultaneously record data streams from brain dynamics, motor behavior, and environmental events, and it requires data-driven analyses methods for multi-modal data to dissociate the brain from non-brain processes (Makeig et al., 2009; Gramann et al., 2011). The main goal of MoBI is to model and understand natural cognition during unrestricted exploratory action in the immediate environment (Gramann et al., 2014; Parada, 2018; Parada and Rossi, 2021).

**FIGURE 2:**
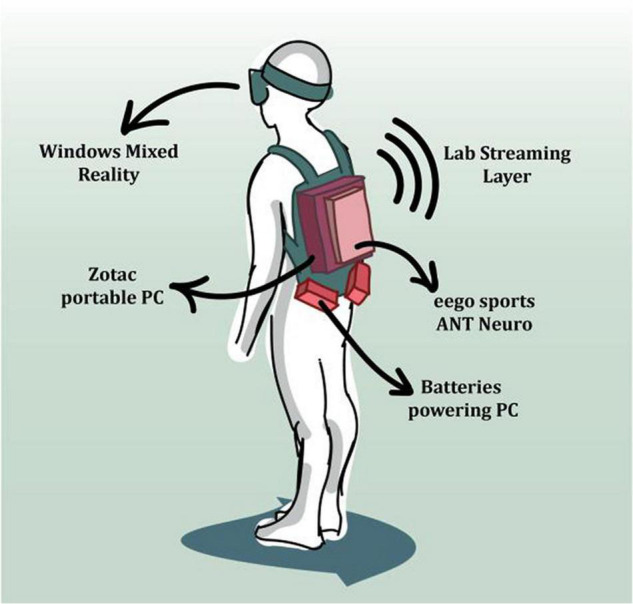
The illustration depicts a MoBI setup using mobile EEG hardware combined with virtual reality and motion capture through the VR tracking system [from Djebbara et al. (2021); used with permission].

*Mobile Imaging* means that participants should be allowed to actively explore the environment in order to reflect the neural dynamics underlying embodied cognitive processes. This necessitates small and lightweight measurement instruments. *Brain/Body Imaging* refers to the investigation of the neural mechanisms of cognitive processes that make use of our physical structure for cognitive goals, and the connection of mind and behavior, perception and action, and sensorimotor coupling on the ecological scale. Both brain and behavioral dynamics have to be recorded in synchrony to explore the bidirectional influence between behavior and brain dynamics. Capturing brain/body dynamics will require multiple sensors to record the different data streams and software to integrate them synchronously (see Figure 2).

Studies in the real world, while providing high ecological validity, do miss control of unwanted factors and cannot simply repeat stimuli material to gain a better signal-to-noise ratio in the signal of interest. Thus, for controlled and repeated stimulus presentation, head-mounted virtual reality (VR) or augmented reality (AR) displays can be integrated in the MoBI hardware system providing an alternative for presenting participants with different environments that can be actively explored while allowing for experimental control and systematically manipulating experimental variables of interest. Furthermore, other stimulus modalities, such as auditory and tactile stimuli, could also be compatible with head-mounted VR displays (Jungnickel et al., 2019).

### Previous Mobile Brain/Body Imaging Studies in Neuro-Architecture

Using MoBI, Banaei et al. (2017) investigated human brain dynamics related to the affective impact of interior forms when the perceiver actively explores an architectural space. The experimental task required participants to naturally walk through different architectural spaces with interior forms extracted from a large corpus of architectural pictures. The rooms represented different combinations of interior forms derived from formal cluster analysis of pictures of the real built environment. Importantly, in order to investigate human brain dynamics related to the affective experience of interior forms during architectural exploration, multimodal data were recorded including EEG and motion capture (Banaei et al., 2017).

The authors found that curvature geometries of interior forms influenced brain activity originating from the anterior cingulate cortex (ACC) while the posterior cingulate cortex and the occipital lobe were involved in the processing of different room perspectives (Banaei et al., 2017). This MoBI architectural neuroscience study demonstrates that both the architectural interior form (such as type, location, scale, and angle) and the exploration of the surroundings will shape the experience of the built environment, providing a neuroscientific basis for architectural design (Banaei et al., 2017). Additionally, this research illustrates the potential of MoBI to investigate human brain dynamics and natural experience of participants actively exploring architectural environments.

Another MoBI study by Djebbara et al. (2019, 2021) investigated the human brain dynamics during transitions through doors of different widths. The authors aimed to investigate how architectural affordances affect brain dynamics by creating three kinds of transitions differing in their passability. Of the three doors, only one did not afford to be transitioned. In the experimental task, implemented in VR, the participants moved from one room to a second room, passing one of the three doors connecting the rooms. The door width which could either be impassable (narrow), passable (medium), or easily passable (wide) formed the operational definition of architectural affordance in their experiment. For priming different interactions with this environment, the authors used a Go/NoGo paradigm either prompting the person to pass through the door (the Go condition), or indicating that the person should not pass through the door (NoGo condition). EEG was used to record their brain activity during the task and a Self-Assessment Manikin (SAM) questionnaire was used to measure participants’ emotional experience after every trial (Djebbara et al., 2019, 2021).

The subjective reports from the SAM showed that different transition affordances influenced the architectural experience of participants. Different door widths influenced participants’ emotional experience, especially when instructed to pass through the door (i.e., forced interaction with the environment) as compared to instructions that did not require interactions with the environment. The physiological results, on the other hand, revealed that brain activity in visual sensory regions and motor areas reflected the affordance of the transition already around 200 ms, irrespective of whether participants knew that they should or should not pass into the second room. This reflects an automated processing of the affordance present in the built environment even if no further interaction with the environment is planned. In addition, differences in the post-imperative negative variation (PINV), a component of the event-related potential (ERP) of the EEG, were visible only in trials that required an interaction with the environment (Go-trials) while in the NoGo condition, this architectural affordance effect was not observed. In other words, the possible interactions with the transition automatically activated cortical areas underlying perceptual and motor responses even in the absence of planned interactions while additional affordance-specific modulations of brain activity were observed during interactions with the built environment (Djebbara et al., 2019, 2021).

The results from Djebbara et al. (2019) support the view that possibilities of imminent actions shape our perception (Djebbara and Gramann, 2022). This view is consistent with the propositions of direct perception and perception-action coupling within ecological psychology (Djebbara et al., 2019; Gepshtein and Snider, 2019). The reasons why imminent action possibilities will influence our architectural perception are that the information is exactly embedded inside imminent action and will further emerge and be perceived during the exploration process rather than a signal transformation, representation, and computation process. Much like Warren’s (1984) research helped elucidate the behavioral dimension of architectural experience, the study of Djebbara et al. (2019) is an exemplary case of integrating the theoretical framework of ecological psychology with neuro-architecture.

In short, MoBI makes it possible to discover, quantify and visualize the embodiment of human agents in an architectural environment with all relevant dimensions of architecture such as aesthetics, ergonomics and more, which can’t be realized by a stationary experimental paradigm. MoBI is an efficient technique to study natural cognition in architectural exploration. However, as the interaction with the environment can become relatively complex in terms of sensory information and motor behavior, a cautious and systematic approach is advisable. As suggested by Parada (2018) and King and Parada (2021), the careful and incremental approach to introducing more complex environments and motor behavior, going from highly controlled setups to more ecologically valid ones, ensures the replicability and control over variables. In other words, by first identifying what to look for, e.g., cortical or behavioral features, in a highly controlled experiment, it is then possible to introduce incremental complexity and assess the quality of the more ecologically valid experiments.

## Conclusion

Although neuro-architecture is a thriving field, there are two methodological limitations within neuro-architectural research (Higuera-Trujillo et al., 2021). The existing research in the field of architectural neuroscience mainly addresses aesthetics out of many different relevant architectural aspects. The brain imaging methods that are typically used require participants to remain stationary, which prevents natural interactions with their architectural surroundings.

In the present article, we argued that concepts of ecological psychology like affordance and active exploration could extend the horizon of the research questions within neuro-architecture to include ergonomics in architecture, which widens the theoretical and empirical framework under which neuro-architectural research is conducted leading to a more comprehensive picture. That is, both the utility and beauty in architecture should be investigated including the analyses of the underlying neural mechanism. Accordingly, inspired by several empirical studies, the operational definition of variables with regards to architectural ergonomics could be established from the perspectives of the complementarity between environmental properties and the agent’s physical capacities, as well as the perception-action loop during architectural exploration. This, however, requires new technological solutions to imaging human brain dynamics during active exploration and interaction with the built environment.

Emerging brain imaging techniques like MoBI, implementing the exploration proposition of ecological psychology in experimental protocols, overcome the limitations of prevalent stationary brain imaging methods and improve the ecological validity of empirical neuroscientific research. Based on the potential of MoBI, more ecologically valid experimental research within the field of neuro-architecture can be conducted. Existing MoBI studies already show evidence of how the brain perceives its surroundings. These new insights can be used to improve architectural design strategies and regulations to eventually improve human health and well-being.

In summary, we described an integrative methodological framework to combine ecological psychology with state-of-the-art neuroscience methods for neuro-architectural empirical research, aiming at extending the horizon of the research questions in the field of neuro-architecture and improving the ecological validity of its experimental framework. This is a promising way to push the field of neuro-architecture forward.

## Author Contributions

All authors listed have made a substantial, direct, and intellectual contribution to the work, and approved it for publication.

## Conflict of Interest

The authors declare that the research was conducted in the absence of any commercial or financial relationships that could be construed as a potential conflict of interest.

## Publisher’s Note

All claims expressed in this article are solely those of the authors and do not necessarily represent those of their affiliated organizations, or those of the publisher, the editors and the reviewers. Any product that may be evaluated in this article, or claim that may be made by its manufacturer, is not guaranteed or endorsed by the publisher.

## Footnotes

1 “Form follows function.”

2 “Less is more.”

3 “There should be no features about a building which are not necessary for convenience, construction or propriety” (Pugin, 1841).
